# Experimental Models to Investigate Viral and Cellular Dynamics in Respiratory Viral Co-Infections

**DOI:** 10.3390/microorganisms13112444

**Published:** 2025-10-25

**Authors:** Ozge Yazici, Claudia Vanetti, Mario Clerici, Mara Biasin

**Affiliations:** 1 Department of Biomedical and Clinical Sciences, University of Milan, 20157 Milan, Italy; ozge.yazici@unimi.it (O.Y.); mara.biasin@unimi.it (M.B.); 2 Department of Pathophysiology and Transplantation, University of Milan, 20122 Milan, Italy; 3 IRCCS Fondazione Don Carlo Gnocchi, 20148 Milan, Italy

**Keywords:** viral co-infections, respiratory viral infections, respiratory viruses, viral interference, host immune responses, experimental models

## Abstract

Respiratory viral co-infections by viruses such as influenza virus, SARS-CoV-2, and respiratory syncytial virus (RSV) are a significant clinical issue in high-risk populations such as children, elderly patients, and immunocompromised individuals. Sequential and simultaneous co-infections exacerbate disease severity, leading to acute respiratory distress syndrome (ARDS), prolonged hospitalization, and increased mortality. Molecular and immunological interactions are complex, context-dependent, and largely unknown. Experimental models of infection that accurately mimic human respiratory physiology are required for the study of viral dynamics, virus–virus interactions, and virus–host interactions. This review outlines a range of complex in vitro and ex vivo models, including organoids, air–liquid interface cultures, lung-on-a-chip platforms, and in vivo animal models, highlighting their ability to simulate the complexity of respiratory co-infections and their limitations. The field has developed significantly, despite challenges like variability across viral strains, timing of infection, and non-standardization of models. Integration of multi-omics technologies and application of highly translational models such as non-human primates and lung-on-a-chip technology are promising avenues to uncover the molecular determinants of co-infection and guide development of targeted therapeutic strategies. Interrelatedness of experimental models and clinical outcomes is highly critical to improve prevention and treatment of respiratory viral co-infections mainly among high-risk populations.

## 1. Introduction

Respiratory viral infections are a severe clinical problem [1,2], particularly in high-risk populations, i.e., children, elderly, and immunocompromised patients [3]. While other types of co-infections, such as bacterial–viral, can also impact disease severity [4,5], this review focuses mainly on viral co-infections. In such viral co-infections, a host may be infected by more than one virus, either sequentially or simultaneously, which can lead to serious complications such as acute respiratory distress syndrome (ARDS), prolonged hospitalization, and increased mortality [6,7,8,9,10,11,12,13]. Moreover, the risk of concurrent infections is increased due to the distinctive characteristics of the respiratory system that make co-circulating viruses highly infectious, but the majority of these co-infections go undetected or misdiagnosed [14]. The SARS-CoV-2 pandemic further exacerbated this scenario [15], altering seasonal cycles of circulation for respiratory viruses and influencing co-infection prevalence rates [16,17,18].

The most challenging aspect of respiratory viral co-infections at the cellular and molecular levels lies in their dynamics. Multiple viruses co-infecting the same host may inhibit and counteract each other’s infectivity, e.g., by inducing antiviral immunity, or show synergistic action, enhancing their replication [19,20]. In addition, viral–viral mode of interaction is dependent on several factors including the involved viruses’ type and strain, order, time of infection, and host immune system status. The interaction of the co-infecting viruses may induce immune dysregulation and lead to cytokine storm [21], altered immune responses [22], and may also impair the function of the epithelial barrier [23]. Such mechanisms lead to a series of unpredictable pathological processes, which are difficult to manage from a clinical point of view.

While clinically relevant, the precise molecular and immunological mechanisms regulating viral respiratory co-infections have been only superficially clarified and need dedicated studies to allow more and more tailored therapies among patients with respiratory viral comorbidities.

This, in turn, outlines the requirement of experimental models that can simulate the real physiological condition and recapitulate viral replication dynamics, host immunity, and host–pathogen interaction. These models enable researchers to deepen the cellular/molecular processes behind viral interference and immune responses, providing insight into the mechanisms that ultimately lead to severe disease. Moreover, these systems may be exploited in the identification of biomarkers that are associated with specific clinical outcomes.

**Figure 1:**
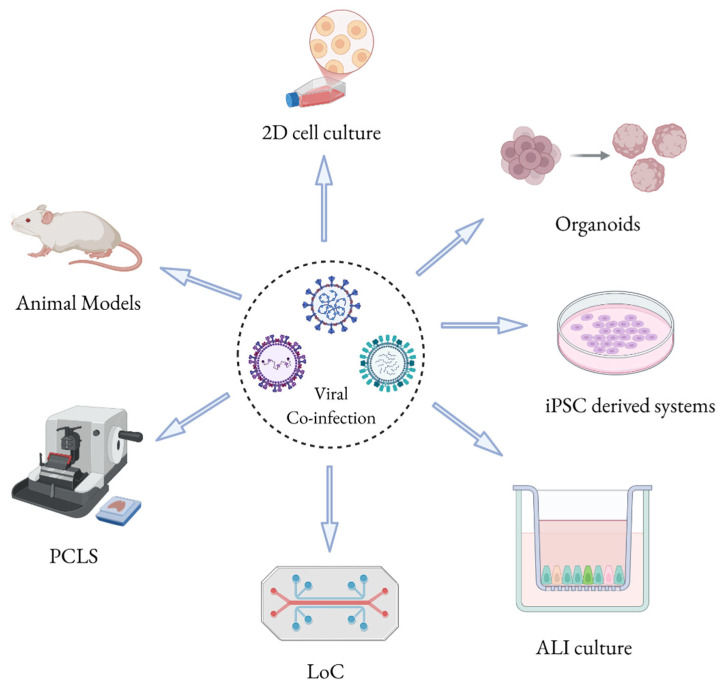
Experimental models employed to study viral co-infections. 2D cell culture, organoids, iPSC (induced pluripotent stem cell)-derived systems, ALI (air–liquid interface) culture, LoC (lung-on-a-chip), PCLS (precision-cut lung slices), and animal models. Created with BioRender.com.

This review summarizes the in vitro, ex vivo and in vivo models that can be used for the investigation of molecular mechanisms induced by specific respiratory viral co-infections. Additionally, it summaries how these models replicate the structure and physiology of the human respiratory system as well as their intrinsic limitations (Figure 1).

## 2. Experimental Models to Study Respiratory Viral Co-Infections

Understanding the complex interactions of viral co-infections requires experimental models that precisely reproduce the structural, cellular, and immunological characteristics of the human respiratory system [24]. At present, different approaches, able to assess the complex interactions among different pathogens in the same host, may be employed. Conventional in vitro models such as immortalized 2D cell cultures still represent the most widespread ones [25]; however, they usually do not accurately mimic the multicellular complexity and physiological relevance of the in vivo state. On the other hand, in vivo models, which more accurately resemble the intricacy of a multicellular organism [26], are costly and need dedicated animal facilities to be managed. There is, therefore, an urgent need for more advanced in vitro and ex vivo models that can mirror virus–virus and virus–host interactions under a physiologically relevant setting. To properly address this claim the scientific community has recently developed a wide range of new models, ranging from 3D organoids and air–liquid interface (ALI) cultures to precision-cut lung slices (PCLS) and lung-on-a-chip (LoC) systems. At the same time, several animal models previously used to study mechanisms of single viral infections have been adapted to investigate co-infections. These tools, extremely helpful in dissecting the molecular mechanisms of respiratory viral co-infections, are emerging as fundamental systems in support of the clinical and public health practice, investigating host responses and allowing the identification of therapeutic targets [26]. These increasingly cutting-edge models are key to enquiry the immunological, molecular, and pathological impact of co-infections, making clear how viruses coexist, compete or collaborate in specific anatomical districts.

### 2.1. 2D Cell Culture Models

2D cell culture models are the most widespread models for studying respiratory viral co-infections [27,28]. They are widely used because of their simplicity, scalability, and the potential to rapidly gain insights into virus–host interactions.

These cellular models allow for the examination of viral replication, immune response, and cellular-level dynamics in vitro [26]. An overview of 2D cell culture models routinely employed to investigate respiratory virus single infection and/or co-infection research is presented in Table 1. In particular, cell lines such as A549, Calu-3, and BEAS-2B [29,30,31,32] recapitulate different aspects of the respiratory epithelium.

A recent study demonstrated how RSV and SARS-CoV-2 co-infection reconfigures homeostasis in A549-hACE2 cells. Indeed, it was demonstrated that following co-infection RSV replication augments and this correlates with (1) a higher expression of a RSV receptor protein, ICAM1; (2) the activation of pro-inflammatory signaling pathways; (3) the disruption of autophagy mechanisms; and (4) an increase in the number and length of cellular conduits. Collectively, these changes exhibited a distinct viral and molecular signature when compared to single infections [33].

Enhanced viral replication during co-infection has been described for other virus pairs. For example, the human parainfluenza virus type 2 (hPIV-2) increases influenza A virus (IAV) replication in Vero E6 cells by promoting syncytia formation and the expression of the hPIV-2 fusion and haemagglutinin-neuraminidase proteins [34]. In A549 cells co-infected simultaneously or one hour apart with IAV and influenza B (IBV) viruses, IBV infection was enhanced [35]. Furthermore, in multiple cell types, co-infection with IAV and SARS-CoV-2 resulted in mutual enhancement of viral replication, through the up-regulation of viral entry receptors [36].

Conversely, the inhibitory effect of IBV on IAV infection rate has been thoroughly studied in MDCK cells [37,38]; similarly, a study by Drori et al. (2020) [39] showed that RSV replication was suppressed by IAV, both in vitro in HEp2 cells and in vivo. This suppression was carried out through the upregulation of interferon-induced proteins (IFITs) [39].

Finally, in another comprehensive study by Haney, J. et al. (2022) [40], a combination of live-cell imaging, scanning electron microscopy, cryo-electron tomography, and neutralization assays demonstrated that co-infection of A549 cells with RSV and IAV resulted in the formation of hybrid virus particles. These particles harbor surface glycoproteins and ribonucleoproteins from both IAV and RSV [40].

These findings illustrate how interactions between viruses can suppress or augment replication dynamics, depending on the pair of viruses [39], and can even lead to unpredictable events [40].

While these models are ideal for high-throughput screening at cell-level research, they have their limitations. First, they lack the immune component which is a driving factor in shaping the disease outcome. Second, they do not display the complexity of in vivo systems, including multicellular interactions and tissue organization. New advances in 2D culture systems, such as co-culture systems with immune cells or genetic engineering, are at least partially bridging this divide [41]. These increasingly complex 2D models enable researchers to model more realistic situations of respiratory viral co-infections providing deeper insight into disease mechanisms and potential therapies.

**Table 1:** 2D Cellular Models Commonly Used in Respiratory Virus Research.

Cell Type	Description	References
**A549** (Human alveolar basal epithelial cells)	Used for studying respiratory viruses such as SARS-CoV-2, and RSV. A549 cells are known for their susceptibility to viral infections and are used in viral co-infection studies.	[33]
**Calu-3** (Human lung adenocarcinoma cells)	Used for studying respiratory viruses, especially SARS-CoV-1, 2 and RSV. Calu-3 cells replicate human lung tissue and are ideal for co-infection models with respiratory viruses.	[29,42]
**BEAS-2B** (Immortalized human bronchial epithelial cells)	Commonly used for respiratory viral studies including RSV, and SARS-CoV-2. BEAS-2B cells mimic bronchial epithelium and are often used to study viral replication and immune response.	[43,44]
**H1299/ACE2** (Human non-small cell lung carcinoma cells)	Use of non-small cell lung carcinoma cell line in respiratory virus research, especially for SARS-CoV-2.	[45]
**Vero** (African green monkey kidney epithelial cells)	Widely used in respiratory virus research, including efficient replication of SARS-CoV-2 and adapted strains of influenza virus.	[46,47]
**LLC-MK2** (Monkey kidney cells)	Used for studying respiratory viruses, including co-infection models for viruses like Human Metapneumovirus (HMPV) and RSV.	[48]
**HEp-2** (Human epithelial cells)	Frequently used for respiratory viral replication studies, such as those involving influenza, RSV, and coronaviruses.	[39,49]
**MDCK** (Madin-Darby Canine Kidney cells)	Commonly used for influenza virus research and respiratory viral co-infection studies. MDCK cells are often employed to study viral replication and virus–host interactions.	[50]
**Huh7** (Human hepatocellular carcinoma cells)	Although primarily used for HCV research, Huh7 cells can be utilized for respiratory viral infections and co-infection models involving influenza or coronaviruses.	[51]
**293T** (Human embryonic kidney cells)	Can be used to study viral replication in respiratory infections, including co-infection studies, due to their ability to support viral gene expression.	[52]
**NCI-H292** (Human pulmonary epithelial cells)	Can be used for respiratory viral co-infection studies, particularly those studying respiratory tract infections.	[53]
**THP-1** (Human monocytic leukemia cell line)	Can be used for studying immune responses in viral co-infection models, especially involving respiratory viruses like influenza and rhinovirus.	[54]
**U937** (Human histiocytic lymphoma cell line)	Can be used to study immune responses during respiratory viral co-infections, often in models involving macrophage activation.	[55]
**HeLa** (Human cervical epithelial cells)	Used for studying viral replication and host responses, including rhinovirus A, and RSV.	[56,57]

### 2.2. 3D Cell Culture Models

Organoids are three-dimensional (3D) cell culture systems derived from pluripotent stem cells (PSCs), adult stem cells (ASCs), and primary tissues or tumor-derived cells that can self-assemble into structures resembling in vivo tissues [58,59,60]. Lung organoids can replicate the cellular components and functionality of the native lung, while maintaining the proper cell to cell and cell to matrix interactions. The regenerative capacity of the stem cells supports organoids’ ability to renew, and make them useful to study lung development, injury, and repair mechanisms. Additionally, these models have the potential to support physiologically relevant platforms for respiratory viral co-infections and are increasingly being recognized as powerful systems for exploring competition, synergistic or antagonistic viral interactions within the same host-like environment.

**Figure 2:**
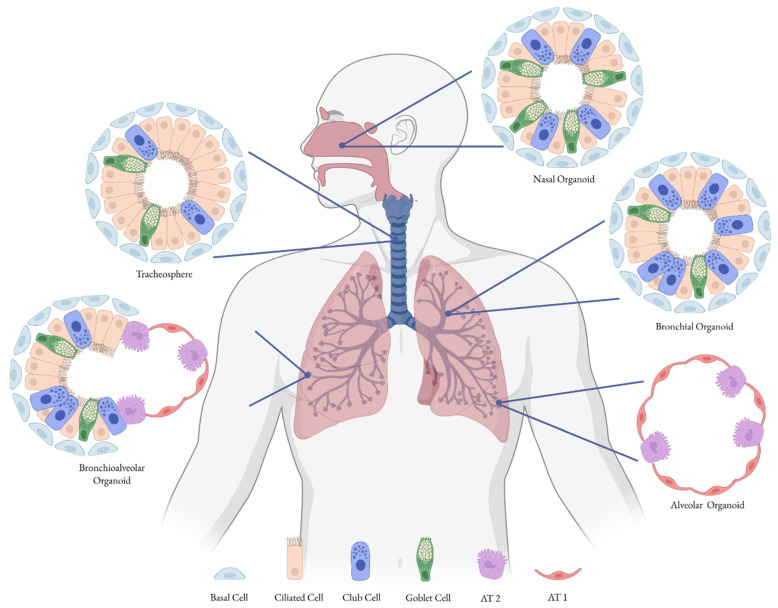
Representative airway and lung organoids. Images of a tracheosphere, a bronchial, a bronchioalveolar, an alveolar organoid and a nasal organoid showing their characteristic cell types. Created with BioRender.com.

As reported in Table 2, lung organoids are classified into four main types based on the structures they represent: alveolar organoids, bronchial organoids, bronchioalveolar organoids, and tracheospheres (Figure 2). However, lung bud organoids are sometimes also included because they are closer to an early lung development stage, rather than a mature structure [61,62]. Bronchial lung organoids mimic smaller conducting airways; therefore, they contain primarily basal, ciliated, and mucous-producing cells [62,63]. In contrast, alveolar organoids resemble the shape of alveoli so contain predominantly AT1 and AT2 cells [62,63]. In addition, human nasal organoids (HNOs) derived from adult or infant nasal stem cells have emerged as a non-invasive model that closely mimics the upper airway epithelium. These HNOs can be differentiated at the air–liquid interface and have been effectively used to study respiratory viruses such as RSV and SARS-CoV-2, revealing age--dependent differences in infection dynamics and immune responses (Figure 2, Table 2) [64,65,66,67].

Overall, patient-derived airway organoids provide a 3D context suitable for studying epithelial-immune interactions during viral exposure. For example, Sachs et al. (2019) demonstrated that airway organoids can be co-cultured with immune cells, such as neutrophils or lymphocytes, to investigate host responses to viral infections like RSV [68]. These iPSC-derived organoids and co-cultures hence would provide a model for complex viral co-infections, especially for understanding patient-specific susceptibilities and drug responses [69,70]. This makes them important tools for studying the molecular and immunological mechanisms behind co-infections, assessing host vulnerability, and discovering new treatment strategies that target multiple pathogens simultaneously [60].

**Table 2:** Organoids Commonly Used in Respiratory Virus Research.

Organoid Type	Description/Origin	Viruses Used for Infection
**Tracheospheres**	Spheroids grown from tracheal stem cells	Used to study differentiation and to assess infectivity of the influenza virus [71].
**Bronchiospheres/Bronchial Organoids**	Derived from progenitor cells of bronchi, mainly basal cells; AT2 cells co-cultured with lung mesenchymal cells	Used to model influenza and SARS-CoV-2 infections [72].
**Alveolar Organoids**	Derived from alveolar progenitor cells (AT2 cells)	Used as a model for respiratory viruses, SARS-CoV-2 [72,73].
**Bronchioalveolar Organoids**	Lung organoids with CHIR99021-induced SCGB1A1^+^ bronchiolar cells	Employed to study infections caused by influenza virus and SARS-CoV-2 [72].
**Lung Bud Organoids**	Derived from hPSCs (mesoderm and pulmonary endoderm); develop into airway organoids	Used in RSV infection studies [74].
**Nasal Organoids**	Generated from human nasal epithelial stem/progenitor cells; recapitulate nasal mucosa structure and function	Used to study SARS-CoV-2 and RSV; valuable for modeling viral entry, replication, and host responses [67,75,76,77]

The application of these sophisticated 3D models has enabled important advances in multiple studies investigating respiratory infections by a single virus [78,79,80,81]. However, only a few studies have exploited this complex model to study the dynamics of co-infections. In particular, Ekanger et al. (2022) developed a human organotypic airway and lung organoid array that faithfully recapitulates bronchiolar and alveolar differentiation, enabling infection with both influenza virus and SARS-CoV-2 [72]. This dual infection model revealed cooperative viral replication. More recently, Kim et al. (2023) [82] leveraged human alveolar type II (hiAT2) organoids to investigate SARS-CoV-2 and influenza A co-infection in a single culture. Results showed that each virus replicated faster in the presence of the other, while a striking cytokine storm, including IL-6, IL-8, TNF, and CCL3, was triggered, mirroring the immune dysregulation characterizing severe COVID-19 cases [82].

Considering their contribution to elucidating the dynamics of individual respiratory virus infections, these models are expected to become widely employed in co-infection research in the near future.

### 2.3. Alternative Cell Culture Models to Study Respiratory Viral Co-Infections

Air–liquid interface (ALI) cultures derived from primary human bronchial epithelial cells have been instrumental in studying viral interactions [83,84]. These cultures are established by isolating primary bronchial epithelial cells from human donors and expanding them under submerged conditions until they reach confluence. Then, the apical surface of the cells is exposed to air while the basolateral surface remains in contact with culture medium. This ALI triggers differentiation of the cells to a pseudostratified epithelium, comprising basal, ciliated, and goblet cells. The resulting tissue closely resembles the in vivo respiratory epithelium [84] (Table 3). One of the key advantages of ALI cultures is their ability to replicate the mucociliary structures and barrier functions of the human airway. Tight connections preserve the integrity of the epithelium while ciliated cells actively transport mucus which traps pathogens [85,86]. For example, a study comparing ALI cultures and airway organoids demonstrated that IAV efficiently infected ALI cultures, replicating typical viral growth kinetics and inducing antiviral cytokine responses, thereby illustrating the model’s relevance for studying single-virus infections in a physiologically realistic frame [87]. In the context of co-infections, Vanderwall et al. (2022) employed bronchial airway epithelial cells (AECs) from children and older adults, differentiated ex vivo at an ALI to generate organotypic cultures [88]. Upon co-infection with SARS-CoV-2 and human rhinovirus (HRV-16), the authors observed that HRV inhibited SARS-CoV-2 replication in an interferon-dependent manner, while SARS-CoV-2 did not reciprocally affect HRV [88]. These findings are consistent with results from other ALI-based models employing primary human airway epithelial cells, where differentiation at the ALI leads to organoids [89,90], and in primary human bronchial epithelial cell-based ALI-culture [91]. Using the same models, SARS-CoV-2 replication was shown to be inhibited or delayed by prior or concurrent infection with other respiratory viruses such as influenza virus [89,92,93,94], RSV [94] and human metapneumovirus [94]. Moreover, rhinovirus has been shown to inhibit IAV replication by inducing interferon-stimulated gene (ISG) expression in primary human airway epithelial cells differentiated at the ALI into organoid cultures [95]. In a similar model, rhinovirus suppressed both influenza A and B replication [96]. Conversely, rhinovirus replication was inhibited by co-infection with influenza A or RSV in a study by Essaidi-Laziosi et al. [97]. Discrepancies among these studies may stem from differences in viral strains and their capacity to induce interferon-mediated antiviral responses.

Additionally, ALI cultures can be genetically manipulated or altered by cytokine exposure to replicate the different disease states, such as COPD or asthma [84,98]. The ability to place immune cells such as macrophages together with epithelial cells in the ALI culture system creates a more physiologically relevant model of viral entry, replication kinetics, virus–host immune response interactions, and virus–virus interactions during co-infections, thus providing insights into disease mechanisms and antiviral drugs [82,99].

ALI-based co-culture systems are also adjustable to incorporate endothelial cells on the basal side of the transwell inserts to have this alveolar-capillary barrier mimicry. Blom et al. (2016) developed a triple co-culture system with epithelial cells (16HBE), macrophages, and dendritic cells to represent the human airway barrier and study immune-modulatory effects, offering likewise usefulness for testing virus-induced epithelial dysfunction and host response [100]. Although limited research has been performed on single-virus and co-infections in this triple co-culture setup, the system provides a physiologically relevant platform to dissect virus–virus and virus–host interactions in human airway model, which will be instrumental for future studies.

iPSC-derived systems serve as an excellent vehicle for studying respiratory viral infections and co-infections because they may be further differentiated into patient-specific lung epithelial, endothelial, and immune cell types. They offer physiological relevance by capturing the individual genetic backgrounds of patients to investigate host–pathogen interactions in a more personalized fashion (Table 3).

iPSC-derived alveolar type II (AT2)-like cells can mature at an ALI, recapitulating key alveolar characteristics such as surfactant protein expression, lamellar-body formation, and functional epithelial barrier properties [101]. Such cells offer a powerful platform to investigate virus–host interactions and could potentially be used to study co-infection dynamics. In this frame, Huang et al. (2020) used iPSC-derived alveolar epithelial type II cells (iAT2s) to model SARS-CoV-2 infection and observed robust viral replication along with a moderate and delayed interferon response [102]. Such models may also support co-infection scenarios, thus shedding light on how sequential or simultaneous infections interfere with host antiviral defenses.

iPSCs can also be used to generate organoid systems. A notable example comes from the previously mentioned study by Kim et al. (2023), which employed human pluripotent stem cell-derived alveolar type II organoids (hiAT2) to investigate interactions between SARS-CoV-2 and IAV [82].

Another approach that could potentially be utilized to investigate respiratory viral co-infection is Precision-cut lung slices (PCLS). These are thin slices of three-dimensional lung tissue with both live cellular and the extracellular matrix components; therefore, are ex vivo models with significant physiological relevance [103]. Harvested lungs from explanted or deceased donors are inflated with low-melt agarose to maintain anatomical structure, followed by vibratome slicing to produce viable PCLS, which retain viability and are functional in oxygenated media for long periods [104]. These models have been previously used to investigate viral infections and innate immune responses in situ [105].

PCLS are robust models for studying viral co-infections in the lungs preserving airway epithelium, alveolar structures, and innate immune cells, although recruitment of circulating immune cells is absent, and endothelial components owing to the significant structural integrity. These systems are suitable to perform both single and co-infections to assess the nature of the viral interactions and may serve as a platform for testing therapeutic interventions such as antivirals and immunomodulatory therapies in circumstances that mimic physiological conditions as close as possible. Moreover, researchers may utilize human donor-derived PCLS to investigate infectious processes and design personalized treatments specific to each patient [104,106,107]. An example of PCLS used to study co-infections comes from a study on swine lung slices, where the co-infection with porcine respiratory coronavirus and two influenza A viruses reduced viral virulence compared to mono-infections [107]. Similar results were observed in a porcine respiratory cell line, where lower viral titers in co-infected samples suggested viral interference between the two RNA viruses [107].

Lung-on-a-chip (LoC) technology provides a groundbreaking way to model human respiratory physiology in vitro and to dynamically mimic breathing movement, the air-blood barrier, and multicellular interactions all within a microengineered system [108]. These microfluidic devices characteristically consist of a porous membrane between two microchannels: one containing human alveolar epithelial cells, exposed to air, and the other containing pulmonary microvascular endothelial cells under fluid flow, both of which together recapitulate key aspects of the alveolar-capillary interface [109] (Table 3).

LoC systems have already been used for modelling single respiratory viral infections and have the potential to be adapted for studies of viral co-infections. For example, Si et al. used a human airway-on-a-chip model of SARS-CoV-2 infection to demonstrate that the infected epithelium released pro-inflammatory cytokines, caused barrier disruption and damage to the endothelial compartment which were found to be hallmarks of severe COVID-19. The chip also allowed testing of antivirals, revealing that amodiaquine inhibited infection while hydroxychloroquine did not, and combination therapy with nafamost at extended the treatment window for influenza A. The airway-on-a-chip can further be adapted to model co-infections by sequentially or simultaneously introducing multiple viruses. Using this system, researchers would be able to track viral dynamics and host responses by simulating a physiologically relevant environment [110].

In addition, strategies have been developed to integrate immune cells, such as macrophages and neutrophils into LoC devices, enabling complex immune-epithelial-endothelial interactions during viral infection. For example, Man et al. described a human lung alveolus-on-a-chip model that incorporated peripheral human monocyte-derived macrophages to model the alveolar-capillary interface under an air–liquid interface with vascular flow. Upon infection with influenza H3N2, the model demonstrated a pronounced decrease in viral titers, due to phagocytosis of infected epithelial cells by macrophages, and yet sustained infection caused macrophage cell death and epithelial junction disruption, contributing to tissue injury. Elevated levels of pro-inflammatory cytokines were detected in the supernatants, circulating immune cells were recruited, and pyroptosis was activated in macrophages. Importantly, blocking pyroptosis with caspase-1 inhibition reduced lung inflammation and injury suggesting the importance of resident immune cells in the modulation of pulmonary response during viral infection [111].

The LoC and other chips may prove valuable for drug screening when accounting for conditions that mimic viral co-infections. As discussed above, this knowledge can provide greater insight into how co-pathogens may potentially alter antiviral efficacy, and whether their presence can contribute to drug-resistance. The surface area, scaling and reproducibility of these platforms can be beneficial for both mechanistic studies and therapeutic testing.

### 2.4. Animal Models

Animal models are a valuable tool for furthering our understanding of respiratory viral co-infections and their complexities, including viral interference, augmented pathogenesis, and immune response modulation. Animal models provide the in vivo context necessary to confirm results from in vitro and ex vivo systems and to evaluate disease outcomes including morbidity, transmission, and tissue pathology.

Murine models are used frequently because of their suitability for genetic manipulation and well-established immune responses and have been used for co-infection models that assess the interaction of two or more respiratory viruses. For example, multiple studies investigated how the sequence of co-infection with IAV and RSV impacts disease severity. In one study, BALB/c mice intranasally inoculated with IAV and RSV 24 h apart, demonstrated higher IAV viral loads at day 7, more airway resistance, decreased lung compliance and increased mortality [112]. Moreover, decreased RSV titers were found in co-infected mice [39]. Conversely, in another study, RSV significantly reduced IAV replication when administered up to 30 days prior, along with substantial differences in cytokine profiles and innate immune cell populations observed in co-infected BALB/c mice compared with single infections [113]. Again, several studies have reported the ability of prior rhinovirus infection to attenuate mouse coronavirus (MHV-1) [114], IAV [115] and pneumonia virus of mice (PVM) [116] lethality. These findings highlight how the order of infection can alter disease outcomes, effects best recapitulated in animal models. Another example comes from a study on RSV/SARS-CoV-2 co-infection [117], which illustrates perfectly how timing of infection influences viral interference, leading to markedly different outcomes. The authors showed that, compared to single infections, RSV/SARS-CoV-2 simultaneous co-infection or RSV followed by SARS-CoV-2 confers protection against SARS-CoV-2-induced disease and reduces its replication. In contrast, SARS-CoV-2, followed by RSV exacerbates SARS-CoV-2 disease, while reducing RSV replication in lung tissue and protecting against RSV-induced pathology.

It is worth pointing out that a major limitation of using animal models, mainly mice, relies in the fact they do not fully reproduce the respiratory clinical outcomes observed in humans. For instance, differences in host physiology, immune responses, and viral tropism can lead to outcomes that do not always mirror those observed in humans. For example, in murine models, RSV typically induces only mild disease and limited pathology, thus affecting the translatability of certain findings [118]. To address this limitation, some studies employ PVM, a natural rodent pathogen that more closely mimics the pathogenesis and clinical manifestations of severe human RSV infection. It has been shown that mice intranasally administered with non-replicating IAV particles before or after PVM infection, were significantly protected against lethal PVM infection [119]. Another example is the use of the mouse hepatitis virus MHV, a group 2 coronavirus, that produces clinical and pathological SARS-like disease [120]. In this context, experimental models of co-infections revealed that a non-lethal pre-infection with MHV-1 protects against IAV and SARS-CoV infections in BALB/c mice [114,121].

To further address these limitations, transgenic mouse models expressing human receptors or immune components are often used. These models better replicate human disease features and improve the relevance of findings. Transgenic mice with human ACE2 (hACE2) have been used to study SARS-CoV-2 co-infection with other viruses. For example, Kim et al. observed a more severe outcome in K18-hACE2 mice initially infected with IAV and 3 days later with SARS-CoV-2. Higher morbidity levels, 100% mortality, increased viral persistence in lung, lymphopenia and impaired adaptive immune responses were reported as well, emphasizing a synergistic mechanism in this co-infection model [21]. Similarly, Bai et al. (2021) reported increased SARS-CoV-2 viral load and more severe lung damage in K18-hACE2 mice co-infected with IAV, in line with their in vitro observations [36]. Finally, it was demonstrated that IAV-infected mice were more susceptible to severe disease when coinfected with SARS-CoV-2 two days later. Notably, prior immunity to influenza, but not to SARS-CoV-2, prevented severe disease and mortality, highlighting the value of seasonal influenza vaccination in reducing the risk of severe influenza/COVID-19 co-infection [122].

Golden Syrian hamsters have been employed to study SARS-CoV-2 and IAV co-infection, as well; both sequential or simultaneous infections induced more severe lung inflammation and systemic weight loss than single infections; intriguingly, when hamsters were first infected with influenza A (H1N1) followed by SARS-CoV-2, SARS-CoV-2 replication was reduced while H1N1 one was increased, highlighting the order-dependent dynamics of viral interference and pathogenesis [123], which were also observed in other studies [124,125]. In addition, researchers found that simultaneous co-infection of female hamsters with SARS-CoV-2 and influenza A (H3N2) reduced IAV replication, but not SARS-CoV-2 [126]. Variability in these results may be attributed to factors such as animal sex, timing of infection, and differences in the influenza virus strain used.

Ferret models are particularly useful to examine co-infections with influenza since they share similarities to human lung physiology and flu symptoms. Ferrets infected with sequential strains of influenza show evidence of viral interference that is dependent on the timing and strain used, with some level of delayed secondary viral shedding [127,128]. Furthermore, primary IAV infection results in significant neutrophil infiltration and changes in host gene expression in a dynamic pattern over the course of infection, supporting the potential for competitive or synergistic interaction in a future live-animal model [129]. Another study comparing ferrets co-infected with SARS-CoV-2 and IAV to those infected with a single virus showed more severe disease in the co-infected animals [130].

Large animal models, such as swine, have also been used to investigate respiratory viral co-infections. For instance, co-infection with porcine parainfluenza virus 1 and influenza A (H1N2) did not exacerbate clinical respiratory signs or viral loads compared to influenza A infection alone, although some differences in lung lesions were observed. This indicates that the pathogenic outcome of co-infections can be virus-specific and not always synergistic [131].

Non-human primates (NHPs) have the most relevant immune and respiratory systems for human modelling of respiratory infections. NHPs are the least used laboratory animals because of ethical, logistical and cost challenges, but they have been used to study several respiratory viral infections [132], using longitudinal assessments of viral kinetics, immune responses as well as lung histopathology. Rhesus macaques, for example, display an immune profile, transient viral shedding and moderate lesion formation in lung tissues resembling human COVID-19 even at immune profiling [133,134].

Moreover, NHP models have been used to study the immune memory that some may gain from infection and protective immunity upon reinfection, wherein prior SARS-CoV-2 infection provides some protection against rechallenge with the same viral strain [135].

Overall, animal models remain indispensable for dissecting the systemic impact of respiratory viral co-infections, including immunopathology, viral synergy, and therapeutic outcomes.

## 3. Discussion and Conclusions

Respiratory viral co-infections pose a complex challenge to human health [24,136,137,138], and generating suitable experimental models to study the biological, immunological, and pathological consequences is therefore crucial. New advancements with both in vitro and ex vivo systems yield more physiologically relevant systems [139], disclosing the dynamics of viral–viral interaction and virus–host interaction, as well as the potential impact that viral co-infections can exert on immune response shaping and disease outcome.

In vivo models are indispensable to further investigate the complex dynamics induced by infections and co-infections in multicellular organisms. Differently from in vitro systems, animal models provide a complete biological system where composite interactions among organs, immune compartments, and pathogens can be evaluated [140]. In vivo models give a systemic view of the co-infection manifestations, permitting a more detailed evaluation of the sequence and timing of viral exposures, and are essential to assess clinical outcomes, including morbidity, mortality, and tissue pathology.

Nevertheless, studies of respiratory viral co-infections based on several of the most advanced ex vivo systems and more sophisticated animal models remain scarce. Of note, systems such as lung-on-a-chip platforms and non-human primate models have not yet been fully explored in this context. Lung-on-a-chip technology, for example, is an in vitro platform that closely mimics key physiological features of the human lung, including tissue-tissue interfaces, mechanical forces, and fluid dynamics [141]. Similarly, at the in vivo level, NHPs represent the most translationally relevant animal model due to their several similarities to humans, including the respiratory anatomy and immunological features [132]. This model has been widely used to study single respiratory viral infections, but, despite a few studies on viruses with different tropism, e.g., SIV/SARS-CoV-2 [142], investigations into respiratory viral co-infections in NHPs are missing.

Currently, experimental models used to explore respiratory viral co-infections are still experiencing several limitations that need to be fixed. For instance, variability in viral strains, inconsistencies regarding most accurate inoculum doses, as well as difficulties in reproducing the timing and sequence of infections are still limiting the translatability of findings. Most models, in particular in vitro systems, are designed to study single pathogens and are not yet optimized to investigate the interactions among multiple viruses. Consequently, there are no standardized guidelines especially regarding cell types, viral input and, ultimately, data interpretation, which can even lead to contradictory results across similar studies. Furthermore, multi-omics approaches, such as genomics, transcriptomics, proteomics, and metabolomics, should be considered for application to respiratory viral co-infection studies. These tools may provide a wider view of the molecular mechanisms of viruses’ interaction.

Future efforts should be directed towards standardizing models already in use and applying more recently developed platforms to study respiratory viral co-infections. Findings from high-throughput systems will be essential not only for understanding the mechanisms underlying viral co-infections, but also for conceiving preventive and therapeutic strategies to address this ongoing challenge.

## Figures and Tables

**Table 3:** Advantages and Disadvantages of Major in vitro Models.

Experimental Model	Advantages	Disadvantages
**2D cell culture models**	Simple to handle and maintain; readily available (for immortalized cell lines); high data reproducibility (for immortalized cell lines); suitable for high-throughput testing	Do not recapitulate in vivo system; lack the immune component; no polarization
**Organoids**	Reproduce the 3D structure, resembling in vivo tissues and host-like environment; replicate the cellular components and functionality of the native lung	Difficult to handle due to sophisticated culture conditions; possibility of reversal of epithelial polarity
**Air–liquid interface (ALI) cultures**	Differentiation in pseudostratified epithelium; able to replicate the mucociliary structures and barrier functions of the human airway; exposure to air allowing gas exchange	Requires extended culture time (minimum 21 days) for epithelial differentiation; differentiated cultures lose functionality and integrity after extended culture periods
**iPSC-derived systems**	Possibility to differentiate into patient-specific lung epithelial, endothelial, and immune cell types, capturing the individual genetic backgrounds	The process of guiding iPSCs to a specific cell type is often inefficient; time-consuming
**Lung-on-a-chip (LoC)**	Tissue-like system combined with electronic sensors; coupled with microfluidic devices, recapitulate key aspects of the alveolar-capillary interface	Limited throughput due to low sample numbers and labor-intensive setup; high technical complexity
**Precision-cut lung slices (PCLS)**	Preserve complex lung architecture, retaining all native cell types and extracellular matrix	Progressive loss of tissue architecture and function occurs during culture

## Data Availability

No new data were created or analyzed in this study. Data sharing is not applicable to this article.
